# Antistress, Adoptogenic Activity of *Sida cordifolia* Roots in Mice

**DOI:** 10.4103/0250-474X.56027

**Published:** 2009

**Authors:** Meera Sumanth, S. S. Mustafa

**Affiliations:** Visweswarpura Institute of Pharmaceutical Sciences, 22nd Main, 24th cross, Banashankari IInd Stage, Bangalore-560 070, India; 1 Advinus Labs, Pune, India

**Keywords:** *Sida cordifolia*, swim endurance test, blood glucose and plasma cortisol

## Abstract

Ethanol extract of roots of *Sida cordifolia* was evaluated for antistress, adaptogenic activity using cold restraint stress and swim endurance in mice. Mice pretreated with extract of *Sida cordifolia* showed significant improvement in the swim duration and reduced the elevated WBC, blood glucose and plasma cortisone.

Stress basically is a reaction of mind and body against change in the homeostasis. Stressors are external, environmental demands placed on us to feel stressed[1], whereas an adaptogen increases the power of resistance against physical, chemical or biological noxious agents. *Sida cordifolia,* Family Malvaceae, commonly known as *Bala*, is an Ayurvedic medicine that is used to treat bronchial asthma, cold and flu, chills, lack of perspiration, head ache, nasal congestion, aching joints and bones, cough and wheezing, and edema. The root infusion is given in nervous and urinary diseases and also in disorders of the blood and bile[2]. *Sida cordifolia* has been reported to posess analgesic, antiinflammatory and hypoglycemic activities as well as hepatoprotective activity[3–5]. The present study was taken up to evaluate antistress, adaptogenic and immunomodulatory activity of the ethanol extract of roots of *Sida cordifolia* in mice.

Roots of *Sida cordifolia* were collected from Native Medicare Charitable Trust, Coimbatore, dried in shade, coarsely powdered and subjected to Soxhlet extraction, using 40% ethanol, at a temperature below 60° for 24 h. The extract was concentrated by distilling the solvent and air-dried (10.2% w/w, brown, solid). The extract was subjected to qualitative phytochemical analysis for presence of various constituents like alkaloids, sterols, sugars, glycosides, phenols and tannins, fixed oils and fats, flavonoids, saponins and gums and mucilages. A solution of the extract (10 mg/ml) was prepared by dissolving it in distilled water for oral administration to animals. A water-soluble powder of *Ashwagandha* (Natural remedies, Bangalore) in a dose of 100 mg/kg *po* was used as reference standard antistress drug[6]. Eight-week old Swiss mice of either sex, weighing 20-25 g, maintained on natural day and night cycle, at a temperature of 25±2^0^, commercial pellet diet (Lipton India, Bangalore) and water *ad libitum* were used in study. Institutional Animal Ethics Committee's permission was obtained before starting the experiments. The nature and extent of the untoward reactions following the administration of extract in graded doses up to 3 g/kg body weight orally, was studied in mice (OECD guidelines 423). For swim endurance test, Swiss mice were divided into 4 groups of 6 animals each. Group I mice, administered only distilled water and not subjected to stress, were ‘control’ animals. Group II mice, administered only distilled water and subjected to stress, were ‘stress control’, Group III mice were administered *Sida cordifolia* extract (SCE) 100 mg/kg orally, using oral gavage, for 7 days. The dose was calculated from LD_50_ of the SCE. Group IV animals were administered water-soluble powder of Ashwagandha 100 mg/kg orally. On 8^th^ day, the animals were allowed to swim till exhausted in a propylene tank of dimension 37×37×30 cm, filled with water to a height of 15 cm. The end point was taken when the animals drowned and swimming time for each animal was noted[7]. For cold restraint stress studies animals were treated in the same manner as described for swim endurance test. On 8^th^ day, animals were individually placed in plastic containers of capacity 350 ml. They were immobilized in their normal position, using adhesive tape. The containers were placed in a cold chamber maintained at 6 to 8^0^ for 2 h. The blood was collected by heart-puncture method, in a heparinised tube and WBC count was done using, blood glucose was determined[7,8] and plasma cortisol level was determined[7,9]. Results are expressed as mean±SEM and data was analyzed by one-way ANOVA, using Graphpad instat. The post-hock analysis was carried out by Dunnet's multiple comparison tests to estimate the significance of difference between individual groups.

Qualitative phytochemical analysis of SCE revealed that it contains alkaloids, carbohydrates, glycosides, triterpenoids and steroids. Acute toxicity studies with extract revealed that LD_50_ is more than a dose of 3 g/kg body weight. As shown in Table 1, SCE improves the swim duration and reduces the elevated WBC, blood glucose and plasma cartisol levels.

**TABLE 1:** EFFECT OF ETHANOL EXTRACT OF *SIDA CORDIFOLIA* ON STRESS

Treatment	Swimming time (m)	Total WBC count (cells/cu mm)	Blood glucose (mg/dl)	Plasma cortisol (μg/100 ml)
Control	-	5620.00±80.63	86.24±3.86	13.04 ± 0.24
Stress Control	419.81±10.55	7126.50±35.73*	137.50±4.43*	21.41±0.56*
*Sida cordifolia*	609.42±12.07	5750.20±34.08**	87.93±3.05**	15.63±0.09**
100 mg/kg				
Ashwagandha	716.17±27.28	5679.20±62.98**	85.85±3.52**	13.97±0.38**
100 mg/kg				
	F=45.011	241.48,	333.33	44.798,

n=6, Values are mean±SEM

* p<0.01 v/s control

** p<0.01 v/s stress control

Mice when forced to swim in a restricted space from which they cannot escape, become immobile after an initial period of vigorous activity, indicating the stress[10]. Pretreatment with adaptogen increase swimming endurance in mice[11]. Mice pretreated with SCE show significant improvement in the swimming time. Cold stress typically increases total leukocyte count, eosinophils and basophils. Plant adaptogen are smooth prostressors which reduce the reactivity of host defense system. The mode of action of adaptogens is basically associated with stress system. Adaptogen increase the capacity of stress to respond to the external signals of activating and deactivating mediators of stress response subsequently[12]. The stress induced increase in total WBC count is decreased by SCE, indicating antistress, adaptogenic activity.

Cortisol is released in response to stress and increased plasma cortisol influences the mobilization of stored fat and carbohydrate reserves, which in turn increases blood glucose level. The increased cortisol levels and increased blood glucose level are reversed by antistress agents[13]. The increase in blood glucose level during stress is also due to suppression of glycogenesis, lipogenesis, increased glycogenolysis, lipolysis, and proteolysis, increased insulin-independent peripheral glucose uptake, and insulin resistance[14]. SCE reduced plasma cortisol level as well as blood glucose level, exhibiting antistress activity. Hence, it can be concluded that *Sida Cordifolia* roots possess antistress, and adaptogenic activity, hence can be categorized as plant adaptogen.

## References

[1] Rice P (1999). Restructuring and the production of occupational stressors in a corporatised ambulance service. Health Soc Rev.

[2] http://en.wikipedia.org/wiki/Country_Mallow.

[3] Kanth VR, Diwan PV (1999). Analgesic, antiinflammatory and hypoglycaemic activities of *Sida cordifolia*. Phytother Res.

[4] Rao KS, Mishra SH (1998). Antihepatotoxic activity of *Sida cordifolia* whole plant. Fitoterapia.

[5] Sutradhar RK, Rahman MA, Ahmad MU, Datta BK, Bachar SC, Saha A (2006). Analgesic and antiinflammatory activities of *Sida cordifolia* Linn. Indian J Pharmacol.

[6] Dhuley JN (2000). Adaptogenic and cardioprotective action of Ashwagandha in rats and frogs. J Ethnopharmacol.

[7] Varley H, Gowenlock AH, Bell M (1984). Practical biochemistry.

[8] Thiebot MH, Martin P, Puech AJ (1992). Animal behavioural studies in the evaluation of antidepressant drugs. Br J Psychiatry Suppl.

[9] Singh N, Verma P, Mishra N, Nath R (1991). A comparative evaluation of some antistress agents of plant origin. Indian J Pharmacol.

[10] Negrao AB, Deuster PA, Gold PW, Singher A, Chrousos GP (2000). Individual reactivity and physiology of the stress response. Neurology.

[11] Kiran Babu S, Gupta P, Bhatia G, Maurya R, Nath C, Palit G (2005). Adaptogenic and anti-amnesic properties of Evolvulus alsinoides in rodents. Pharmacol Biochem Behavior.

[12] Tache Y, Selye H (1976). Shift in adenohypophyseal activity during chronic intermittent immobilization of rats. Neuroendocrinol.

[13] Meera S, Mustafa SS (2007). Antistress, adoptogenic and immunopotentiating activity of roots of *Boerhaavia diffusa* in mice. Int J Pharmacol.

[14] Dominiczak B (1999). Glucose homeostasis and fuel metabolism. Medical Biochemistry.

